# The Effects of Rheum Tanguticum Polysaccharide on the Polarization of Th1 and Th2 Cells in TNBS-Induced Colitis in Murine

**Published:** 2005-06

**Authors:** Li Liu, Qi-Bing Mei, Zhi-Peng Wang, Yu-Mei Zhou, Hua Zhang, Yin Long, Jia-Yun Liu

**Affiliations:** 1 *Department of Pharmacology, Fourth Military Medical University, Xi’an, China;*; 2 *North China Pharmaceutical Group Corporation, Shijiazhuang, China*

**Keywords:** rheum tanguticum, polarization of T cell, TNBS

## Abstract

Inflammatory bowel diseases (IBDs) are chronic inflammatory intestinal disorders that are characterized by thickened colon walls, colon ulcerations, including two forms of ulcerative colitis (UC) and Crohn’s disease (CD). UC and CD share some similar clinico-pathological characteristics but their causes are opposite. The imbalance in cytokinesis produced by Th1 and Th2 cells, the subunit of CD4^+^ T cells, plays a key role in the development of IBDs. Although traditional treatment for IBDs is effective to some patients, it has numerous adverse-effects such as immuno-depression. In our previous study we found some therapeutic effects of Rheum tanguticum polysaccharide (RTP) on CD. Our present investigation focuses on the comparison of the effects of RTP (200 mg·kg^-1^, once a day for five days) on UC induced by trinitrobenzene sulfonic acid (TNBS)/ethanol in BALB/c mice and CD induced by TNBS in SD rats. The mechanism of RTP was investigated by using immuno-histochemistry, Elisa assay, flow cytometry and western-blot analysis. Our results showed that RTP had significant therapeutic effects on both UC and CD. The ulcerative index and colon weight were significantly attenuated after RTP treatment while MPO activity in RTP-treated animals was markedly lower than that in the animals of TNBS administration (*P*<0.05, *P*<0.01). RTP also showed significant inhibitory effects on the expansion of CD4+T cells simultaneously improving the imbalance of Th1 and Th2 polarization (*P*<0.01). In conclusion, RTP appears to poses all the pre-requisites to be applied in therapeutic intervention, thus, offering a hope for effective treatment for IBDs.

## INTRODUCTION

Inflammatory bowel diseases (IBDs), including ulcerative colitis (UC) and Crohn’s disease (CD), are complex immune-mediated diseases characterized by chronic inflammation of the intestine (1). In recent decade, UC and CD were considered as more frequently-occurring disease placing big burden on patients and people with sedentary habits (increasingly observed in office personnel). With a prevalence of about 0.1% in many developed countries, the incidence of the disease has increased rapidly in China. It is reported that inappropriate CD4^+^T cell response in mucosa immunity plays an important role in IBDs (2). In addition, the imbalance in cytokines produced by Th1 and Th2 cells, the subunit of CD4^+^ T cells, plays a key role in the development of IBDs (3). Although UC and CD show some similar clinico-pathological characteristics, their causes are opposite (4). Growing evidence has suggested that in humans, there is an excessive Th1 cell response associated with excessive IFN-γ and IL-12 secretion in CD cases, whereas excessive Th2 cell response is associated with excessive IL-4 and IL-10 secretion in UC cases (1). The most common conventional therapy for both UC and CD in clinical practice is the use of anti-inflammation drugs including steroidal and non-steroidal anti-inflammatory drugs, such as 5-amino salicylic acid and glucocorticosteroids (GCs). Although both of them are very effective in short term, they show some side effects, especially GCs have some immunosuppressive effects, which restrict their clinical use (5). Chinese people have used rhubarb as a folk remedy for gastrointestinal disease for two thousand years. Some traditional Chinese decoction contains rhubarb, which has some significant therapeutic effects on both CD and UC in clinic. In our previous studies, the polysaccharide extracted from Rheum tanguticum, one breed of rhubarb, showed striking protective effects on CD induced by TNBS in rats (6). However, there is no report about the effects of Rheum tanguticum polysaccharide (RTP) on UC and the therapeutic mechanism of RTP on CD and UC is still unclear. Elson *et al* (7) once reported that the characteristics of UC and CD could be illustrated by intestinal inflammatory models induced by trinitrobenzene sulfonic acid (TNBS) in different animal strains. Our present study is based on his reports to study the effects of RTP on CD and UC (CD was induced by TNBS/ethanol in SD rats while UC was induced in BALB/c mice) and to explore the effects of RTP on the expansion of CD4^+^T cells and the polarization of Th1 and Th2 cells.

## MATERIALS AND METHODS

### Animals

Adult Sprague-Dawley (SD) rats, weighing 220 g to 250 g, and adult BALB/c mice, weighing 18 g to 20 g, were obtained from the Animal Center of the Fourth Military Medical University and the animals used in the experiment were approved by the Institutional Ethical Committee of the Fourth Military Medical University. They were fed with a standard laboratory diet and tap water ad libitum and kept in a room with controlled temperature (22 ± 1°C), humidity (50-70%), and a 12:12 h light-dark cycle. The total number of animals used three times in the experiment was 34 rats and 57 mice, which were divided into three groups respectively, including saline, TNBS alone, and TNBS + RTP.

### Regents

2,4,6-trinitrobenzene sulfonic acid (TNBS), fucose, galacturonic, galactose, mannos, arabinose, sorbose, glucose and glucuronic standard monosaccharide, ethylenediaminetetraacetic acid (EDTA), dextrans, xanthine and xanthine oxidase from Sigma (St. Louis, MO, USA), hexadecytrimethyl-ammonium from Fluka (Buchs, Switzerland) were used in the experiment. CD4 Mab was purchased from Immunotech Company, and INF-γ and IL-4 were obtained from BioSource International (Camatillo, CA, USA).

### Preparation of polysaccharide from R tanguticum

Rhubarb was identified as Rheum tanguticum Maxim. ex Regel Tolygonaceac by Professor Ren Yi in Northwest University in Xi’an, China. Rheum tanguticum polysaccharide (RTP) was extracted according to the methods described previously (8). In brief, Rheum tanguticum Maxim.ex Regel (1 kg) was fragmented and boiled with ethanol to remove the components dissolved in ethanol. The residue was boiled with water to extract polysaccharides. The polysaccharide-enriched fractions were obtained by precipitation with 5 volumes of ethanol 5 times. After the proteins were removed with freezing-thawing and savage methods, 7 gram of crude Rheum tanguticum polysaccharide from per 100 gram rhubarb was obtained by dialysis (molecular weight cut-off 8000) with distilled water for 72 hours, by concentration and by lyophilization. The gel column (5 cm × 100 cm) was packed with Sephacryl-S400 and eluted with 0.1 N NaCl at a flow rate of 2 ml/min at room temperature. 10 ml elute was collected in a Pharmacia LKB Superfrac fraction collector. Then the polysaccharide contents were determined by the phenol sulphuric acid assay, and the uronic acid contents were used with colorimetrical assay. The molecular weight of RTP was identified by gel chromatography, in which the gel column (1 cm × 80 cm) was packed with Sephacryl-S400 and eluted with 0.1N NaCl at a flow rate of 1ml/min at room temperature. The molecular weight of RTP was 60-80 kDa, which was calculated from the standard MW-eluted volume relationships that were generated with five known different MW dextrans (2 × 10^5^, 3 × 10^5^, 5 × 10^5^, 6 × 10^5^, and 8 × 10^5^). Monosaccharide composition was identified by gas chromatography (GC) (Shimadzu, Japan) with flame ionisation detector (Hewlett-Packard, Vienna, Austria). RTP was composed of galactose, galacturonic, arabinose, glucose, glucuronic and sorbose.

### Induction of experimental inflammatory bowel disease in murine

The experimental CD in SD rats and UC in BALB/c mice were induced with reference to what Elson *et al* (7) described, respectively. The animals were fasted for 24 hours before experimentation but had free access to drinking tap water. After the animals were slightly anesthetized with diethyl ether, TNBS (30 mg TNBS/40% ethanol to rats, 3 mg TNBS/38% ethanol to mice) was slowly injected into the colon through the rectum using a polyethylene catheter (10 cm long and 2 mm external diameter for rats, 6 cm long and 1 mm external diameter for mice). RTP was orally administrated to the animals once a day for 5 days and then they were sacrificed on 6th day after RTP treatment. The distal colon was removed, opened and rinsed thoroughly in ice-cold saline. The lesion area was measured using a 1 mm^2^ grid by a single observer who was blind to treated and untreated animals. The distal colons with the length of 8 cm of rats and 4 cm of mice were weighted. The ratio of colon weight to body weight was used to assess the degree of colon edema, which reflected the severity of colonic inflammation. The samples were excised, and the colonic mucosa was carefully scraped off with a glass slide and snap-frozen in liquid nitrogen, and stored at -80°C to further assay myloperoxidase (MPO) activities within a week. Then the spleen and thymus were removed and weighted.

### Measurement of myeloperoxidase (MPO) activity in the colonic mucosa

MPO activity was determined with a modified method described by Krawisz *et al* (9). In brief, 50 mg colonic mucosa was homogenized with a homogenizer for 40s in an ice-cold 50 mM phosphate-buffered saline solution containing 0.5% hexadecyl- trimethylammonium bromide (pH6.0). The homogenate was freeze-thawed three times, followed by repeated sonication for 30 s. The supernatant was collected after centrifugation (11,000 g, 4°C, 20 min). The change in absorbance at 460 nm was measured spectrophotometrically from 30 s to 90 s, and MPO activity was defined as degrading 1 μmol of hydrogen peroxide per minute at 25°C and expressed as unit per gram of tissue.

### Immunohistochemical analysis on CD4+ T lymphocyte

Immuno-histochemical staining was performed on paraffin-embedded sections by using biotinylated anti-CD4 mAb according to SBC immuno-histochemical kit procedure.

### Flow cytometry on peripheral blood CD4+ lymphocyte

500 μl peripheral blood was taken from caudal vein of SD rats and BALB/c mice, and was diluted by PBS. Immuno-fluorescence labeling was done with anti-CD4. The sample was centrifuged three times at 1,100 g for 5min. This procedure was followed by haemolysis, which was achieved by short incubation with 0.83% NH4Cl pH7.2. The cells were resuspended in 0.1% formaldehyde solution. Finally, flow cytometry was performed with Beckman FASC.

### Western-blot analysis on CD4+ lymphocyte

SD rats and BALB/c mice with IBDs induced by TNBS/ethanol were sacrificed. The lumenal lymphoid node was separated and homogenized in 1 ml lysis buffer in ice bathing for 10 min, then centrifuged at 11,000 g for 5 min at 4°C, and 30 μl suspension was moved to 15 ml lodding buffer. The samples were boiled for 10 min and subjected to electrophoresis on 120 g/L SDS-acrylamide gel. The gel was then electroblotted onto ultrocellulose membrane, and the detection of CD4 protein was conducted by using anti-CD4 mAb.

### Measurement of cytokine production

SD rats and BALB/c mice with IBDs induced by TNBS/ethanol were sacrificed and their mesenteric lymph nodes were removed. Lymph nodes were free of adipose tissue and gently pressed through a wire mesh. Lymphocytes were collected by centrifugation (1,000 g, 10min) and washed twice with RPMI culture medium. Lymphocytes (5 × 10^6^) were cultured in 24-well flat bottomed culture plates in RPIM culture medium containing 10% (v/v) FCS. After 24 h the plates were centrifuged and the culture medium was carefully removed. Cytokine concentrations were measured by using Elisa assay according to manufacturer’s instruction.

### Statistical analysis

The SPSS software was used to determine the statistical significance of the difference. Results were expressed as mean ± standard error. Differences between the two groups were examined using the Student’s t-test. Mortality was examined using c2-test. *P* value with less than 0.05 was considered significant.

## RESULT

### Effects of RTP on TNBS-induced IBDs in murine

**Figure 1:**
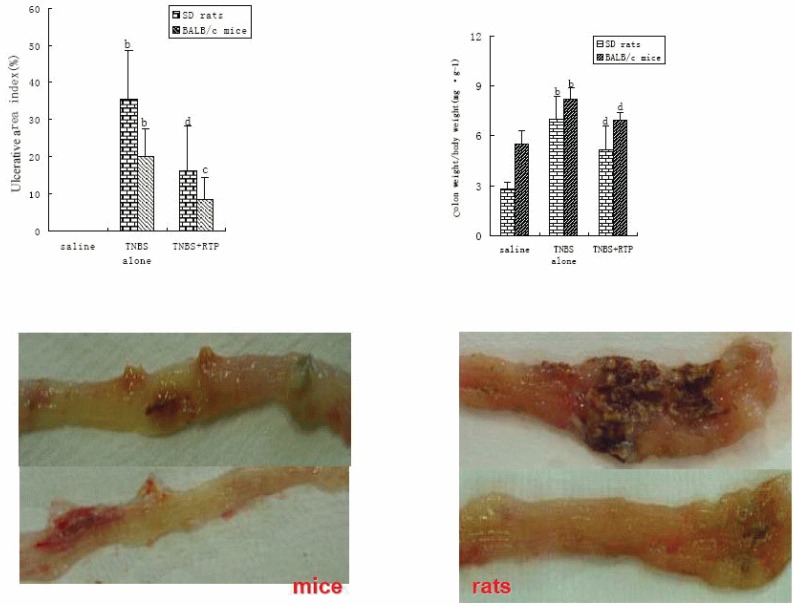
Effects of RTP on TNBS-induced IBDs in murine (n=10, X ± s) Animals were administrated with saline, TNBS in ethanol (TNBS alone) and RTP (TNBS + RTP 200 mg•kg^-1^). The percentage of ulcerative area indicated the ratio of the ulcerative area to total area of colon at 8cm length in rats and at 4cm length in mice. Values were means ± standard error for 10 rats and 10 mice in each group. ^a^*P*<0.05 vs saline, ^b^*P*<0.01 vs saline; ^c^*P*<0.05 vs TNBS alone, ^d^*P*<0.01 vs TNBS alone; ^f^*P*<0.01 vs SD rats.

The mortality of BALB/c mice, nearly 70%, was much higher than that of SD rats. The inflammation in BALB/c mice was primarily limited to the colon, whereas the inflammation in SD rats was throughout the gastrointestinal tract and much more severe. The colon damage in SD rats was related to Th1 cell response, which enhanced the immune response so the inflammatory response became obvious. However, Th2 cell response depressed the immune response so the inflammation healing was promoted. RTP presented striking therapeutic effects on TNBS-induced inflammatory bowel diseases in both SD rats and BALB/c mice (Table 1, Figure 1).

**Table 1:** Effects of RTP on survival rate in TNBS-induced IBDs in murine

Animals	Saline	TNBS alone	TNBS + RTP

SD rats	100 (10/10)	71.4 (10/14)	100 (10/10)
BALB/c mice	100 (10/10)	33.3 (10/30)	58.8 (10/17)

### Effects of RTP on MPO activity of colonic mucosa in TNBS-induced IBDs in murine

**Figure 2:**
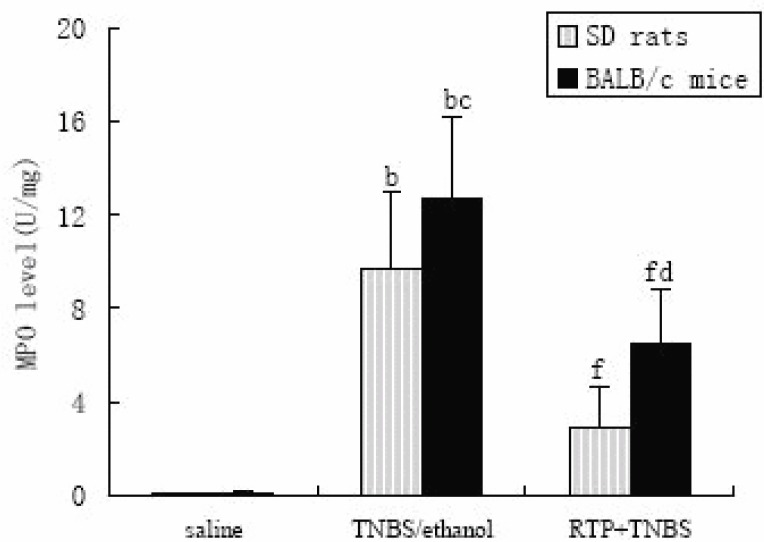
Effects of RTP (200mg·kg^-1^, p.o) on MPO activity of colonic mucosa in TNBS-induced IBDs in murine.

MPO enzyme was used as a quantitative index of inflammation and a marker of neutrophil infiltration in the tissue (9). The MPO activity in the colonic tissue dramatically increased after TNBS-enema. The MPO activity in BALB/c mice was much higher than that in SD rats, which partly explained the cause of higher mortality of BALB/c mice. Treatment with RTP (200 mg·kg^-1^, p.o) significantly inhibited the increase of MPO activity both in SD rats and BALB/c mice (Figure 2).

The colonic mucosa MPO activity was measured in murine-received saline, TNBS in ethanol (TNBS alone) or RTP (TNBS + RTP). Values were means ± standard error for 10 rats and 10 mice in each group. ^a^*P*<0.05 *vs* saline, ^b^*P*<0.01 vs saline, ^c^*P*<0.05 vs the same parameter in SD rats, ^d^*P*<0.01 vs the same parameter in SD rats; ^f^*P*<0.01 vs TNBS/ethanol.

### Effects of RTP on thymus and spleen weight in TNBS-induced IBDs in murine

It was found in the experiment that atrophy of thymus was found in both rats and BALB/c mice after TNBS-enema, whereas spleen was augment in SD rats, atrophy in BALB/c mice, which indicated different immune state in IBDs. The mice administrated with RTP regained spleen weight but RTP did not have any effect on thymus (Table 2), which was related to the increase of thymus or spleen T cells in the early stage of inflammation. Then during the inflammation development T cells migrated to the colon and infiltrated in the local inflammatory site, which induced the dysfunction of thymus and spleen (10).

**Table 2:** Effects of RTP on thymus and spleen weight in TNBSinduced IBDs in murine (n=10, $\bar{X}$ ± s)

Drugs	Spleen Index		Thymus Index	
	BALB/c Mice	SD Rats	BALB/c	MiceSD Rats

Saline	8.3 ± 0.8	2.3 ± 0.4	1.4 ± 0.7	2.4 ± 0.03
TNBS alone	5.0 ± 0.3^b^	3.2 ± 0.4^a^	1.2 ± 0.6	0.9 ± 0.3^b^
TNBS + RTP	6.1 ± 1.2^c^	3.0 ± 0.4	1.1 ± 0.2	1.2 ± 0.5

a *P*<0.05 vs saline;

b *P*<0.01 vs saline;

c *P*<0.05 vs TNBS/ethanol.

### Effects of RTP on CD4^+^T cell expansion in TNBS-induced IBDs in murine

**Figure 3:**
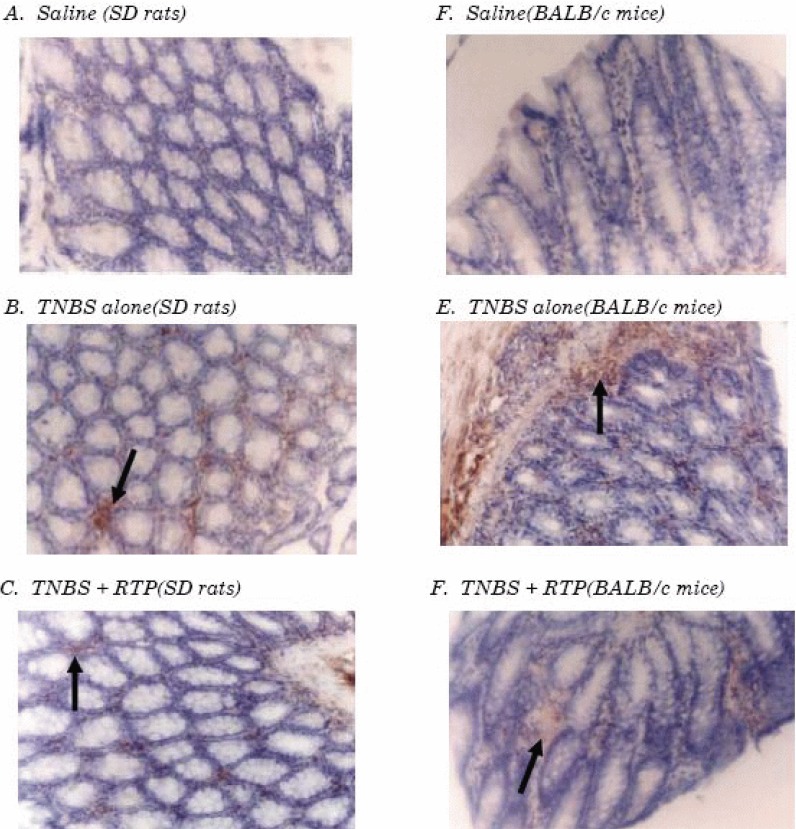
Immuno-histochemical analysis of CD4+ lymphocytes isolated from colon tissue Immuno-histochemical staining of paraffin, colon mucosa sections from SD rats (A, B and C) and colon mucosa sections from BALB/c mice (D, E and F). The sections from the animals treated with saline (A and D), TNBS-induced IBDs (B and E) and RTP treatment (C and F). These photos stained with CD4 antibody showed positive cells (arrow). (Magnification 400×).

**Figure 4:**
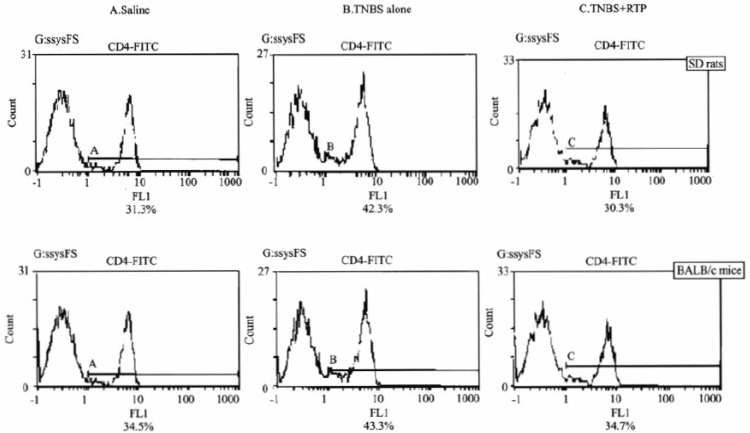
Flow cytometry on CD4+T cell expansion in peripheral blood CD4+T cells significantly increased in both SD rats and BALB/c mice treated with TNBS(B). However, CD4+T cells markedly decreased after RTP administration (C).

**Figure 5:**
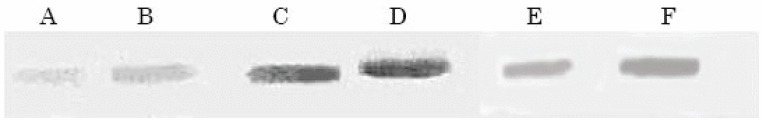
Western-blot analysis Western-blot analysis of CD4 protein from the SD rat (Lane A, C and E) and the BALB/c mouse (Lane B, D and F) lymphoid nude. The lanes from left to right represented the protein isolated from animals administrated with saline (Lane A and Lane B), TNBS /ethanol (Lane C and Lane D) and TNBS + RTP (Lane E and Lane F), respectively.

Immuno-histochemistry, flow cytometry and western-blot analysis were employed to investigate the effects of RTP on CD4^+^T cell expansion. It was found that CD4^+^T cell infiltration in colon inflammation, expansion in peripheral blood, and CD4 protein expression up-regulation were showed in animals suffering from intestinal inflammation and that the resistance of RTP on the expansion of CD4+T cell was presented significantly in both SD rats and BALB/c mice (Figures 3, 4 and 5) (Table 3).

**Figure 6:**
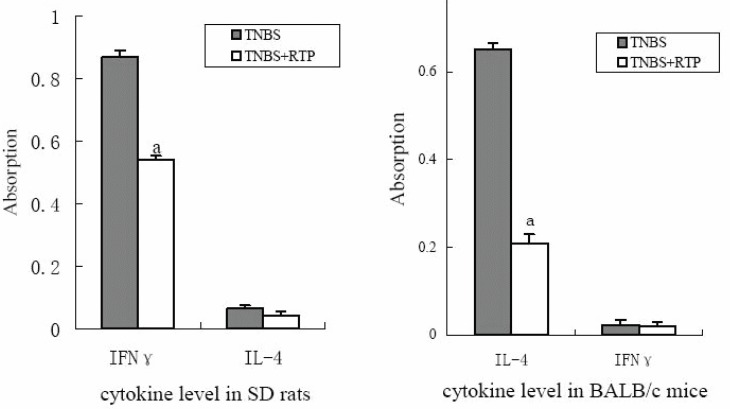
Effects of RTP on cytokine level.

**Table 3:** Effect of RTP on CD4+T cell expansion in peripheral blood (n=6, $\bar{X}$ ± s)

Animals	Saline	TNBS alone	TNBS + RTP

SD rats	31.5 ± 2.16	41.8 ± 3.22	31.9 ± 1.18
BALB/c mice	33.7 ± 1.13	42.9 ± 2.20	35.1 ± 3.24

### Effects of RTP on cytokine secreted by Th1 or Th2 cells in TNBS-induced IBDs in murine

IFN-γ and IL-4 concentration were measured to investigate the effects of RTP on cytokine secretion and the polarization of Th1/Th2 in IBD animals treated with RTP. The product of IFN-γ from SD rats significantly increased but little amount of Il-4 product was detected. The marked decrease of IFN-γ product was observed after RTP treatment. However, Il-4 product from BALB/c mice with UC was much high, whereas IFN-γ product was hardly detected. Il-4 product significantly decreased after RTP treatment in BALB/c mice.

## DISCUSSIONS

In the past few years, a large body of evidence has been accumulated to suggest that the pathogenesis of IBDs involves genetic susceptibility, immunity dysfunction, and interaction among local environment microbes (11). Crohn’s disease (CD) and ulcerative colitis (UC) are together known as inflammatory bowel disease. CD is characteristic of multifucal and transmural inflammation throughout the gastrointestinal tract, whereas UC is primarily limited to the colon. The difference between CD and UC may be related to the imbalance between Th1 cell response and Th2 cell response (3). Although CD and UC share some similar clinico-pathological properties, the causes are opposite. Up to now, there is still no miracle drug to cure the patients. Glucocorticoid is used as main remedy for IBDs, but the severe adverse effects, such as immuno-depression, limit its clinical use. It is urgent to develop new therapeutic strategies. In our previous study, we found Rheum tanguticum polysaccharide (RTP) showed a striking therapeutic effect on CD of rats (6). In our present research, we found some significant therapeutic effects of RTP on both UC and CD. The failure of TNBS/ethanol to induce UC in CD4-/- mice indicated that CD4+T cells played a key role in the development of IBD (12). It was also found that RTP had some significant effects on the resistance of the expansion of CD4 +T cells in periphery blood, the infiltration of CD4 +T cell in colon mucosa, and up-regulation of CD4 protein expression, which suggested that the mechanism of RTP on IBDs might be related to modulate CD4+T cell dysfunction. The data indicated that RTP might have potential therapeutic effects on IBDs.

The IBD animal model induced by TNBS is inexpensive and easy to duplicate so it is the most common model used to screen anti-IBD drug. Therefore, TNBS/ethanol-induced animal model is suitable for estimating the therapeutic effects and mechanism of RTP on IBDs. In 1995, Neurath, *et al* (13) reported that different inflammatory characteristics of IBDs were illustrated in TNBS/ethanol-induced intestinal inflammation animal models in different strains. The characteristics of Th1 cytokines were similar to human CD, while the characteristics of Th2 cytokines were similar to human UC. In our present study, the mucosa inflammation induced by TNBS was more severe in SD rats than that in BALB/c mice. Five days after TNBS administration to the rats, the colon showed widespread mucosal necrosis and gross ulceration, whereas the mortality was much high in BALB/c mice. Th1 cells promoted the development of inflammation and immune response. However, Th2 cells promoted inflammation healing by depressing immune response (14). It was proved in this experiment that TNBS induced different inflammatory responses in different animal strains. RTP showed significant effects on the two forms of IBDs (UC and CD) and improved Th1/Th2 polarization towards the balance of cytokine secretion.

The balance of Th1 and Th2 cells plays an important role in normal immune response. The abnormal mucosa immune system displays an unbalanced response consisting of either excessive Th1 response or inadequate Th2 response (3). The polarization of Th1 and Th2 cells is affected by some cytokines, but the mechanism is unclear. Cytokines secreted by Th1 cells, such as TNF-γ and IL-12, may promote Th1 cell polarization while IL-4 and IL-10 secreted by Th2 cells may promote Th2 cell polarization. Recently, some monoclonal antibody of cytokines has been found to regulate the imbalance of cytokine. For example, an antibody of TNF-γ has been used in clinical trial to treat the patients with moderate or severely active CD.

In conclusion, the results obtained in the present study showed significant effects of RTP on laboratory animals with UC induced by trinitrobenzene sulfonic acid (TNBS)/ethanol in BALB/c mice and CD induced by TNBS in SD rats. There was marked decrease of both IFN-γ in CD rats and IL-4 concentration in UC mice. It appears that the therapeutic effects of RTP on IBDs are related not only to the resistance of expansion of CD4+T cells but also to the improvement of the balance of polarization of Th1 and Th2 cells. Therefore, RTP may be considered as a potential phyto-therapeutic treatment in IBDs.
